# Evolution of Chatbots in Nursing Education: Narrative Review

**DOI:** 10.2196/54987

**Published:** 2024-06-13

**Authors:** Fang Zhang, Xiaoliu Liu, Wenyan Wu, Shiben Zhu

**Affiliations:** 1 Department of Science and Education, Shenzhen Baoan Women's and Children's Hospital, Shenzhen, China; 2 Medical Laboratory of Shenzhen Luohu People’s Hospital, Shenzhen, China; 3 School of Nursing and Health Studies, Hong Kong Metropolitan University, Hong Kong, China

**Keywords:** nursing education, chatbots, artificial intelligence, narrative review, ChatGPT

## Abstract

**Background:**

The integration of chatbots in nursing education is a rapidly evolving area with potential transformative impacts. This narrative review aims to synthesize and analyze the existing literature on chatbots in nursing education.

**Objective:**

This study aims to comprehensively examine the temporal trends, international distribution, study designs, and implications of chatbots in nursing education.

**Methods:**

A comprehensive search was conducted across 3 databases (PubMed, Web of Science, and Embase) following the PRISMA (Preferred Reporting Items for Systematic Reviews and Meta-Analyses) flow diagram.

**Results:**

A total of 40 articles met the eligibility criteria, with a notable increase of publications in 2023 (n=28, 70%). Temporal analysis revealed a notable surge in publications from 2021 to 2023, emphasizing the growing scholarly interest. Geographically, Taiwan province made substantial contributions (n=8, 20%), followed by the United States (n=6, 15%) and South Korea (n=4, 10%). Study designs varied, with reviews (n=8, 20%) and editorials (n=7, 18%) being predominant, showcasing the richness of research in this domain.

**Conclusions:**

Integrating chatbots into nursing education presents a promising yet relatively unexplored avenue. This review highlights the urgent need for original research, emphasizing the importance of ethical considerations.

## Introduction

Nursing education, crucial for positive patient-professional relationships [1,2] and continuous professional development [3], holds a pivotal position in global health care systems [4], driving progress [5] and integrating technological advancements to enhance patient-centered care [6,7]. A study on oncology nursing provided compelling evidence for nurses, addressing challenges and advocating for specialized education and safety measures in the escalating global cancer burden [8]. A recent meta-analysis of 12 studies with 821 participants evaluated the role of virtual reality in nursing education, which revealed substantial enhancements in knowledge but identified no distinguishable disparities in skills, satisfaction, confidence, and performance time, underscoring the imperative for additional investigations in these domains [9]. Another study explored the usability and feasibility of extended reality smart glasses in core nursing skill training for undergraduate students, uncovering positive effects on engagement, learning satisfaction, and competency improvement and highlighting the potential of smart glasses as an impactful educational strategy in nursing training [10]. However, nursing education encounters obstacles such as a worldwide scarcity of nursing expertise [11], uneven distribution of resources [12], potential disparities between theoretical and practical aspects [9], restricted interdisciplinary collaboration [13], insufficient opportunities for professional development [14], and the ramifications of the global COVID-19 pandemic [15].

In the swiftly evolving landscape of artificial intelligence (AI) and smartphone proliferation, the integration of large language models such as ChatGPT into chatbots is emerging as a trend, with chatbots progressively showcasing the potential to revolutionize mental health [16], behavior [17], and knowledge [18] within the dynamic and advancing field of deep learning. Recent studies on education have accentuated the use of chatbots to deliver personalized learning experiences [19,20] by tailoring content delivery to the unique needs of individual students, thereby augmenting comprehension and retention. Concurrently, chatbots provide an easily accessible platform for continuous learning [21], affording students the opportunity to retrieve information at their convenience and cultivating a culture of self-directed learning. Moreover, the interactive attributes of chatbots facilitate real-time feedback, permitting the prompt rectification of misconceptions and fostering a more profound grasp of intricate health care concepts [22]. The adaptability of chatbots caters to diverse learning styles, ensuring inclusivity in education [23]. Despite these advantages, few studies investigate the integration, development, and feasibility of chatbots within nursing education.

Our aim is to meticulously investigate and amalgamate the existing literature pertaining to the integration of chatbots in nursing education by reviewing selected articles. By scrutinizing studies sourced from 3 prominent databases (PubMed, Embase, and Web of Science), we highlight insightful perspectives on the evolving role of chatbots in nursing education. Approaching this investigation with the perspective of a reviewer, we seek to contribute a nuanced and well-supported analysis of the existing literature on this topic.

## Methods

### Search Strategy

We devised pertinent search queries concerning nursing education and chatbots, with the designated search terms detailed in Section 1 in Multimedia Appendix 1. A thorough investigation encompassing 3 databases—PubMed, Embase, and Web of Science—was carried out from their individual inception dates to November 16, 2023.

### Eligibility Criteria for Study Inclusion

The eligibility criteria were devised in accordance with the PICOS (Population, Intervention, Comparison, Outcome, and Study Design) framework [24]. The study inclusion criteria were meticulously outlined to ensure the accuracy and relevance of the selected research. The specified population comprised nurses or nursing students, including managers and clinical nurses, with a deliberate exclusion of doctors and other professional personnel. The intervention criteria encompassed any chatbot intervention, including chatbot apps, messaging, and web-based interventions, while excluding interventions not specifically focused on chatbots or lacking communication with them. The comparator conditions involved conventional education methods, such as face-to-face or drug interventions, excluding the integration of chatbot interventions. The exclusion criteria also considered comparators that included chatbot interventions at comparable rates but with differing frequencies. The outcomes of interest included results relevant to nursing education, covering levels of medical knowledge, nurses’ engagement with chatbots, and the improvement of practical skills. The study design inclusion criteria accepted any design. Detailed eligibility criteria are shown in Section 2 in Multimedia Appendix 1.

### Selection Process and Outcomes of Interest

The search findings were imported into Covidence (Veritas Health Innovation) while adhering to established protocols. The screening process involved 2 stages. Initially, titles and abstracts were screened, followed by a thorough review of full-text articles. Duplicated papers were removed using Covidence prior to the screening stages to ensure the integrity of the selection process. Three authors (SZ, XL, and WW) independently and in duplicate executed all screening stages and data extraction, resolving any discrepancies through consultation with the senior author (FZ). To ensure precision and uniformity in data, we formulated a comprehensive data extraction form (SZ and WW) that underwent subsequent refinement (SZ and FZ), in alignment with guidelines from the *Cochrane Handbook for Systematic Reviews of Interventions* [25]. Before full extraction, the form underwent a pilot test on a subset of included studies. Extracted details from all included studies (SZ, XL, and WW) included elements such as publication details (study ID, title, and year), author particulars (lead author contact information), study specifics (country, study design, and objectives), and conclusions.

### Study Design and Statistical Analysis

This was a narrative review. After the screening process, we successfully gathered comprehensive data, encompassing publication details (study ID, title, and year), author particulars (contact information for the lead author), study specifics (country, study design, and objectives), and conclusions. Subsequently, we categorized this data based on the respective year, country, and study design. To provide a visual representation of the trends observed, we conducted percentage calculations for each category. These percentages were then used to illustrate the trend over time and to convey the distribution of studies across various categories.

## Results

**Figure 1.**
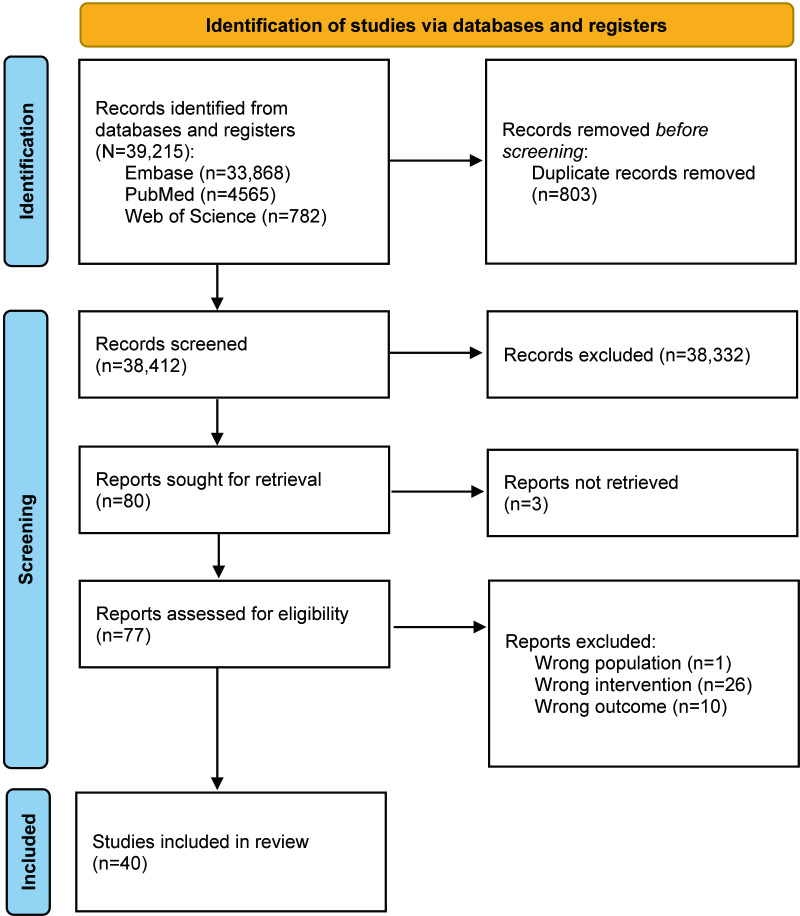
PRISMA (Preferred Reporting Items for Systematic Reviews and Meta-Analyses) flow diagram showing the study selection process.

In total, 38,412 distinct records were identified. Subsequently, an eligibility assessment was conducted on 77 full-text articles, with 3 articles not retrieved, as depicted in Figure 1. Out of these, 37 were subsequently excluded, resulting in the inclusion of 40 articles that met the eligibility criteria for synthesis [26-65].

**Figure 2.**
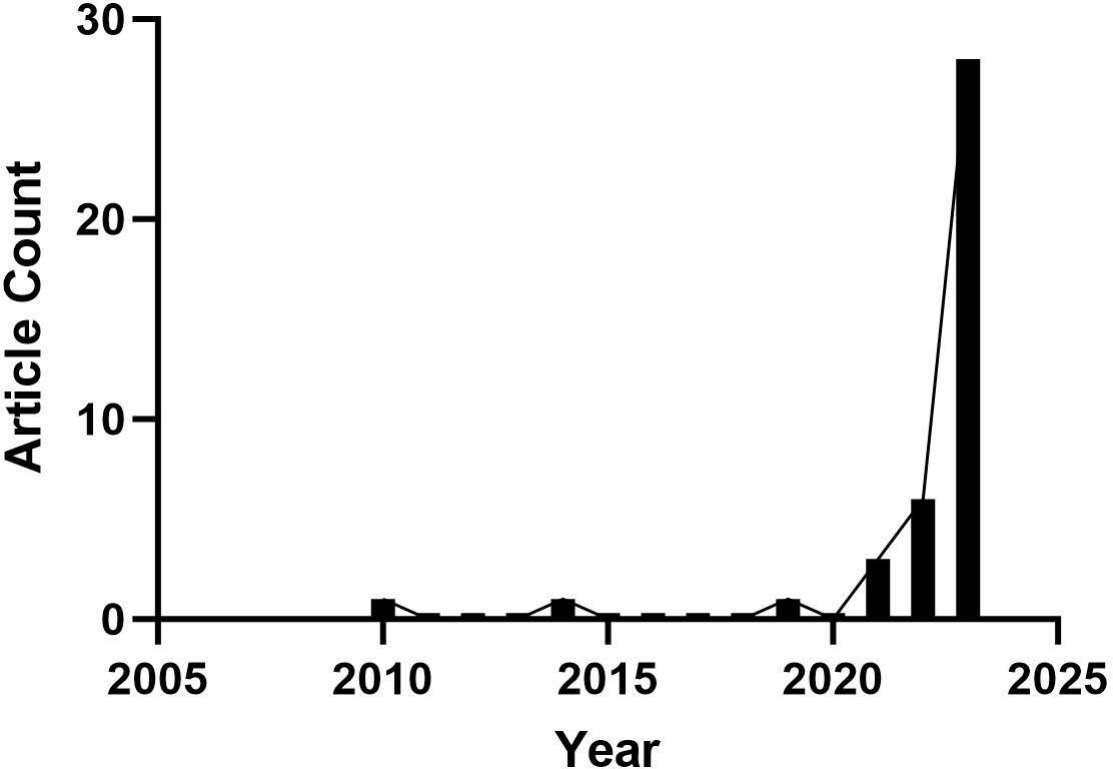
Temporal fluctuations in articles published from 2010 to 2023.

Between 2010 and 2020, on average, 1 article was published every 3‐4 years, culminating in a total of 3 articles, contributing to 8% of the 40 publications. However, a noticeable upswing occurred in 2021, with the publication of 3 (8%) articles. In 2022, the count increased to 6 (15%) articles. The most notable surge transpired in 2023, with the publication of 28 articles, accounting for a substantial 70% of the total publications (Figure 2).

Taiwan province contributed 20% (8/40) of the total articles [31,32,34,35,37,42-44]. Following closely was the United States, contributing 15% (6/40) of the articles [39,40,46,52,55,59]. South Korea secured the third position, representing 10% (4/40) of the articles [41,47,48,63]. Canada [26,28,30], Mainland China [33,50,64], and Singapore [57,58,60] each contributed 8% (3/40) of the articles. Turkey [29,62] contributed 5% (2/40) of the articles. Other countries such as Australia [45], France [38], Germany [49], Hong Kong [36], India [56], Iraq [27], Italy [51], Japan [61], Malta [54], the United Kingdom [53], and Ukraine [65] each contributed 3% (1/40) of the articles.

In our review, the predominant study design was reviews, constituting 20% (8/40) of the total articles [36,46,49,50,56,59,60]. Reviews exemplify a meticulous synthesis of existing literature, providing comprehensive insights and analyses on specific topics. Editorials, comprising 18% (7/40) of the articles [28,39,45,47,52-54], serve as platforms for commentary, opinions, or perspectives on current issues and developments in the field. Commentaries constituted 10% (4/40) of the articles [26,30,35,64], offering critical reflections, analyses, or perspectives on specific subjects. Letters to the editor, making up 8% (3/40) of the articles [27,29,61], provide readers with a platform to express opinions, raise concerns, or offer feedback on published content. Quasi-experimental studies comprised 8% (3/40) of the articles [41,48,58], employing experimental methods without random assignment. Constituting 5% (2/40) of the articles, teaching tips offer valuable insights into effective educational strategies [34,55]. Randomized controlled trials (RCTs), considered the gold standard in experimental design, constituted 5% (2/40) of the articles [42,62]. Experimental design, symbolizing systematic investigation, was embodied in 3% (1/40) of the articles [31]. Empirical articles, grounded in observations and experiences, constituted 3% (1/40) of the articles [32]. Phenomenological studies, delving into lived experiences and perceptions, comprised 3% (1/40) of the articles [33]. Proof-of-concept studies, showcasing the feasibility of an idea or approach, constituted 3% (1/40) of the articles [38]. Mini reviews, furnishing concise overviews, comprised 3% (1/40) of the articles [65]. Descriptive qualitative studies, concentrating on detailed exploration, accounted for 3% (1/40) of the articles [40]. Experimental studies, engaging in controlled testing, made up 3% (1/40) of the articles [43]. Systematic reviews, characterized by methodical literature synthesis, represented 3% (1/40) of the articles [44]. Articles centering on experimentation methodology represented 3% (1/40) of the articles [51]. Development studies, exploring the creation of new methodologies or tools, constituted 3% (1/40) of the articles [57]. Lastly, articles classified as communications, conveying crucial information or updates, represented 3% (1/40) of the articles (Table 1) [63].

**Table 1.** Overview of the extracted studies.

Study	Title	Country	Study design	Year
Abdulai and Hung [26]	Will ChatGPT Undermine Ethical Values in Nursing Education, Research, and Practice?	Canada	Commentary	2023
Ahmed [27]	The Impact of ChatGPT on the Nursing Profession: Revolutionizing Patient Care and Education	Iraq	Letter to editor	2023
Archibald and Clark [28]	ChatGTP: What Is It and How Can Nursing and Health Science Education Use It?	Canada	Editorial	2023
Berşe et al [29]	The Role and Potential Contributions of the Artificial Intelligence Language Model ChatGPT	Turkey	Letter to editor	2023
Castonguay et al [30]	Revolutionizing Nursing Education Through AI Integration: A Reflection on the Disruptive Impact of ChatGPT	Canada	Commentary	2023
Chang et al [32]	Promoting Students’ Learning Achievement and Self-Efficacy: A Mobile Chatbot Approach for Nursing Training	Taiwan	Empirical article	2022
Chang et al [31]	Chatbot-Facilitated Nursing Education: Incorporating a Knowledge-Based Chatbot System Into a Nursing Training Program	Taiwan	Experimental design	2022
Chan et al [64]	Critical Reflection on Using ChatGPT in Student Learning: Benefits or Potential Risks?	China	Commentary	2023
Chen and Kuo [34]	Applying the Smartphone-Based Chatbot in Clinical Nursing Education	Taiwan	Teaching tip	2022
Chen et al [33]	Need Assessment for History-Taking Instruction Program Using Chatbot for Nursing Students: A Qualitative Study Using Focus Group Interviews	China	Phenomenological study	2023
Cheng [35]	Transformation in Nursing Education: Development and Implementation of Diverse Innovative Teaching	Taiwan	Commentary	2021
Choi et al [36]	Chatting or Cheating? The Impacts of ChatGPT and Other Artificial Intelligence Language Models on Nurse Education	Hong Kong	Review	2023
Chuang et al [37]	The Design and Application of a Chatbot in Clinical Nursing Education	Taiwan	Review	2021
Daniel et al [38]	Answering Hospital Caregivers’ Questions at Any Time: Proof-of-Concept Study of an Artificial Intelligence–Based Chatbot in a French Hospital	France	Proof-of-concept study	2022
Teixeira da Silva [61]	Is ChatGPT a Valid Author?	Japan	Letter to editor	2023
Dave et al [65]	ChatGPT in Medicine: An Overview of Its Applications, Advantages, Limitations, Future Prospects, and Ethical Considerations	Ukraine	Mini review	2023
de Gagne [39]	The State of Artificial Intelligence in Nursing Education: Past, Present, and Future Directions	United States	Editorial	2023
Friedman and Goldschmidt [40]	Let Me Introduce You to Your First Virtual Patient	United States	Descriptive qualitative study	2014
Han et al [41]	Analysis of the Effect of an Artificial Intelligence Chatbot Educational Program on Non-Face-to-Face Classes: A Quasi-Experimental Study	South Korea	Quasi-experimental study	2022
Hsu and Chen [43]	Personalized Medical Terminology Learning Game: Guess the Term	Taiwan	Experimental study	2023
Hsu [42]	Mastering Medical Terminology With ChatGPT and Termbot	Taiwan	RCT^a^	2023
Hwang et al [44]	How Artificial Intelligence (AI) Supports Nursing Education: Profiling the Roles, Applications, and Trends of AI in Nursing Education Research (1993‐2020)	Taiwan	Systematic review	2022
Irwin et al [45]	What is ChatGPT and What Do We Do with It? Implications of the Age of AI for Nursing and Midwifery Practice and Education: An Editorial	Australia	Editorial	2023
Johnson et al [46]	When To Err Is Inhuman: An Examination of the Influence of Artificial Intelligence-Driven Nursing Care on Patient Safety	United States	Review	2023
Jung [47]	Challenges for Future Directions for Artificial Intelligence Integrated Nursing Simulation Education	South Korea	Editorial	2023
Kang et al [48]	Awareness of Using Chatbots and Factors Influencing Usage Intention Among Nursing Students in South Korea: A Descriptive Study	South Korea	Quasi-experimental study	2023
Krüger et al [49]	ChatGPT: Curse or Blessing in Nursing Care?	Germany	Review	2023
Liu et al [50]	The Application of Chat Generative Pre-trained Transformer in Nursing Education	China	Review	2023
Mascitti et al [51]	COACH BOT - Modular e-Course With Virtual Coach Tool Support	Italy	Experimentation methodology	2010
Miao and Ahn [52]	Impact of ChatGPT on Interdisciplinary Nursing Education and Research	United States	Editorial	2023
O'Connor [53]	Open Artificial Intelligence Platforms in Nursing Education: Tools for Academic Progress or Abuse?	United Kingdom	Editorial	2023
Scerri and Morin [54]	Using Chatbots Like ChatGPT to Support Nursing Practice	Malta	Editorial	2023
Seney et al [55]	Using ChatGPT to Teach Enhanced Clinical Judgment in Nursing Education	United States	Teaching tip	2023
Sharma and Sharma [56]	A Holistic Approach to Remote Patient Monitoring, Fueled by ChatGPT and Metaverse Technology: The Future of Nursing Education	India	Review	2023
Shorey et al [57]	A Virtual Counseling Application Using Artificial Intelligence for Communication Skills Training in Nursing Education: Development Study	Singapore	Development Study	2019
Shorey et al [58]	Evaluation of a Theory-Based Virtual Counseling Application in Nursing Education	Singapore	Quasi-experimental study	2023
Sun and Hoelscher [59]	The ChatGPT Storm and What Faculty Can Do	United States	Review	2023
Tam et al [60]	Nursing Education in the Age of Artificial Intelligence Powered Chatbots (AI-Chatbots): Are We Ready Yet?	Singapore	Review	2023
Uslu and van Giersbergen [62]	The Effects of Manikin-Based and Standardized-Patient Simulation on Clinical Outcomes: A Randomized Prospective Study	Turkey	RCT	2023
Ye et al [63]	Development of a Chatbot Program for Follow-Up Management of Workers’ General Health Examinations in Korea: A Pilot Study	South Korea	Communication	2021

a RCT: randomized controlled trial.

## Discussion

### Principal Findings

In this paper, we comprehensively examined the temporal trends, international distribution, study designs, and implications of chatbots in nursing education to map the challenges and issues to address in the future. Our analysis highlights significant findings, including a marked increase in research publications in 2023, reflecting growing interest in this field. Contributions from Taiwan province, the United States, and South Korea illustrate the global scope of chatbot integration in nursing education. The diverse study designs reviewed, ranging from reviews and editorials to quasi-experimental studies, indicate the extensive research exploring chatbots’ role in enhancing personalized instruction, patient-care simulations, and critical thinking. Despite these advancements, challenges such as the need for rigorous original research, funding, ethical considerations, and resource distribution disparities remain. Furthermore, addressing these issues through international collaboration and targeted research will be crucial for fully realizing the potential of chatbots in nursing education.

AI language models such as chatbots have caused a revolution in nursing education through the provision of personalized and interactive learning activities. Chatbots are implemented in nursing education for personalized instruction, patients-care simulation, and critical thinking enhancement. Chatbots in health care are used for teleconsultation to improve communication skills, support clinical judgment, and enable remote patient monitoring. Chatbots are a key component in addressing the global shortages of knowledge and resources in nursing training. They bridge theoretical and practical aspects, thereby illustrating the potential of this technology to revolutionize learning processes and change the face of health care services and education.

This study aims to shed light on the evolution of chatbots in nursing education through data analysis of temporal trends. The PRISMA (Preferred Reporting Items for Systematic Reviews and Meta-Analyses) flow diagram facilitates a systematic search procedure, which guarantees a transparent and strict methodology. Indeed, articles published in 2023 accounted for 70% (28/40) of the included articles, which might be due to either increased scholarly interest or intensified effort. This study tries to delve into the technological education aspect of health care, which is a rapidly expanding area. Consequently, it will provide a comprehensive reflection of the dynamic and developing educational sector.

This study provides a new approach about how AI and mobile communication can be applied in and influence nursing education. Chatbots and AI integration can be seen as a technical invention with thrilling effects on mental health, behavior, and knowledge in relation to the field of deep learning. The analysis stresses the sole benefits of chatbots in education, that is, chatbots provide the capacity for individualized learning [27,31,32,39,43,44,47,48,50,51,53,56,60]. The studies focus on problems in nursing education that involve the shortage of global knowledge, condition differences, and lack of relationship between theory and practice [29,35,45,49,58] and illustrate the ways chatbots can cope with these issues.

A detailed study of the worldwide distribution and categorization of chatbot research on nursing education is carried out with reference to international contexts, highlighting major contributions. The participation of United States and South Korea is notable, and Taiwan province has the largest share, accounting for 20% (8/40) of all articles. This regional perspective highlights the universal nature of adding chatbots to nursing education. As the research methodology analysis reveals, reviews cover 20% (8/40) of the articles, providing exhaustive summaries of the present literature. A diverse range of designs that includes commentaries, quasi-experimental studies, teaching tips, and RCTs explains the extensive and varied research on chatbots in nursing education.

In spite of the huge benefits, there are some barriers that nursing education will face as they try to incorporate chatbots. Original research such as RCTs or cohort studies is the most important part of confirming the efficiency of conversational bots. Funding research about advanced techniques and the application of rigorous process need high levels both of staff and finance. The integrity and the security problems of chatbots that provide wrong advice are highlighted, demonstrating the need for correcting the technical problems in order to ensure ethical and secure operations. Funding should be set aside to close resource distribution disparities [39,40,47,57-59], so that students from disadvantaged backgrounds can also have an opportunity to have access to technologically advanced educational resources. Collaboration among those in the academic, technical, and health care disciplines is indispensable as an effort to develop supportive surroundings for the application of chatbots to nursing education globally.

This study demonstrates the substantial changes that chatbots bring into nursing education to make nursing practice more enjoyable. This integration aims at resolving several issues, including the lack of competitiveness from a global perspective and economic disparity, in essence to establish an integrated and dynamic learning environment. Analyzing the small components of chatbots and conducting research on the feasibility, pros, and cons are necessary aims for the future of education [44]. The lack of original research forces us to rely more on the already existing qualitative studies such as commentaries and editorials. Above all, great attention should be given to privacy and ethics when integrating current technologies into the health care education system.

There are some limitations. First, the study only provides a description of the changes over time in articles related to chatbots in nursing education, as well as the distribution of regions and types of articles. Due to the lack of original studies, it does not show the characteristics of papers included in the final analysis. Second, there is uncertainty about whether the specific research topics related to chatbots in nursing education are consistent between countries. Third, there is a lack of in-depth quantitative exploration and discussion regarding the specific application directions of chatbots in nursing education, preventing the formulation of more constructive recommendations.

### Conclusion

Integrating chatbots into nursing education presents a promising yet relatively unexplored avenue. This review highlights the urgent need for original research, emphasizing the importance of ethical considerations. This exploration contributes to the evolving landscape of technology in health care education, bridging gaps and fostering a learner-centric approach aligned with contemporary health care demands.

## Supplementary material

10.2196/54987 Multimedia Appendix 1 Search strategies and eligibility criteria for study inclusion.

## Abbreviations

AI: artificial intelligence
PICOS: Population, Intervention, Comparison, Outcome, and Study Design
PRISMA: Preferred Reporting Items for Systematic Reviews and Meta-Analyses
RCT: randomized controlled trial
