# Supplementary material for: Evolution of Chatbots in Nursing Education: Narrative Review
Source: JMIR Med Educ. 2024 Jun 13;10:e54987. doi: 10.2196/54987 (PMC11186796; doi:10.2196/54987)
Supplement: Multimedia Appendix 1 [file mededu-v10-e54987-s001.docx]

**Multimedia Appendix 1**

**Table of Contents**

[**Section 1: Search strategies** 3](#_Toc150806577)

**[Section 2: Eligibility criteria for study inclusion](#_Toc150806578)** [9](#_Toc150806578)

# **Section 1: Search strategies**

**PubMed Search**

Date of search:16/11/2023

| # | Search | Results |
| --- | --- | --- |
| 1 | "Education, Nursing"[Mesh] OR “nurs* skill” OR “nurs* knowledge” OR “nurs* training” OR “nurs* learning” OR “informatic* nurs*” OR “nurs* study” OR “nurs* teaching” OR “nurs* student” OR “nurs*” OR “pupil nurse” OR “midwifery” OR “nurse academic” OR “nurse educator” OR “nurse Teacher” | 1,167,802 |
| 2 | "chatbot*" OR "chat?bot*" OR "chatter bot*" OR "chatterbot*" OR "chatter robot*" OR "bot*" OR "chat*" | 121,775 |
| 3 | "conversational bot*" OR "conversational agent*" OR "conversational system*" OR "conversational interface*" OR "conversational AI" OR "conversational agenc*" OR "conversational assistant*" | 1,225 |
| 4 | "smart bot*" OR "smartbot*" OR "smart-bot*" | 15 |
| 5 | "dialog system*" OR "dialogue system*" OR "dialogue agent*" OR "dialog agent*" OR "digital assistant*" | 1,299 |
| 6 | "ai agent*" OR "artificial agent*" OR "assistance technolog*" OR "embodied agent*" OR "relational agent*" OR "virtual agent*" OR "virtual assistant*" OR "virtual coach*" OR "virtual character*" OR "virtual human" OR "intelligent agent*" OR "interactive agent*" OR "artificial intelligence" | 83,543 |
| 7 | "social robot*" OR "virtual advisor*" OR "avatar*" OR "infobot*" OR "animated character*" | 3,227 |
| 8 | #1 AND (#2 OR #3 OR #4 OR #5 OR #6 OR #7) | 4,565 |

**Embase Search**

Date of search:16/11/2023

| # | Search | Results |
| --- | --- | --- |
| 1 | ‘Education, Nursing’/exp OR (“nurs* skill” OR ‘nurs* knowledge’ OR ‘nurs* training’ OR ‘nurs* learning’ OR ‘informatic* nurs*’ OR ‘nurs* study’ OR ‘nurs* teaching’ OR ‘nurs* student’ OR ‘nurs*’ OR ‘pupil nurse’ OR ‘midwifery’ OR ‘nurse academic’ OR ‘nurse educator’ OR ‘nurse Teacher’):ab,kw,ti | 700,719 |
| 2 | ('chatbot*' OR 'chat?bot*' OR 'chatter bot*' OR 'chatterbot*' OR 'chatter robot*' OR 'bot*' OR 'chat*'):ab,kw,ti | 7,343,253 |
| 3 | ('conversational bot*' OR 'conversational agent*' OR 'conversational system*' OR 'conversational interface*' OR 'conversational ai' OR 'conversational agenc*' OR 'conversational assistant*'):ab,kw,ti | 506 |
| 4 | ('smart bot*' OR 'smartbot*' OR 'smart-bot*'):ab,kw,ti | 25 |
| 5 | ('dialog system*' OR 'dialogue system*' OR 'dialogue agent*' OR 'dialog agent*' OR 'digital assistant*'):ab,kw,ti | 1,525 |
| 6 | ('ai agent*' OR 'artificial agent*' OR 'assistance technolog*' OR 'embodied agent*' OR 'relational agent*' OR 'virtual agent*' OR 'virtual assistant*' OR 'virtual coach*' OR 'virtual character*' OR 'virtual human' OR 'intelligent agent*' OR 'interactive agent*' OR 'artificial intelligence'):ab,kw,ti | 50,988 |
| 7 | ('social robot*' OR 'virtual advisor*' OR 'avatar*' OR 'infobot*' OR 'animated character*'):ab,kw,ti | 3,487 |
| 8 | #1 AND (#2 OR #3 OR #4 OR #5 OR #6 OR #7) | 91,902 |
| 9 | #8 AND ([embase]/lim NOT ([embase]/lim AND [medline]/lim) OR ([embase classic]/lim NOT ([embase classic]/lim AND [medline]/lim))) | 33,868 |

**Web of Science Search**

Date of search:16/11/2023

| # | Search | Results |
| --- | --- | --- |
| 1 | TI=("Education*, Nursing" OR "Nursing Education" OR “nurs* skill” OR “nurs* knowledge” OR “nurs* training” OR “nurs* learning” OR “informatic* nurs*” OR “nurs* study” OR “nurs* teaching” OR “nurs* student” OR “nurs*” OR “pupil nurse” OR “midwifery” OR “nurse academic” OR “nurse educator” OR “nurse Teacher”) OR AB=("Education*, Nursing" OR "Nursing Education" OR “nurs* skill” OR “nurs* knowledge” OR “nurs* training” OR “nurs* learning” OR “informatic* nurs*” OR “nurs* study” OR “nurs* teaching” OR “nurs* student” OR “nurs*” OR “pupil nurse” OR “midwifery” OR “nurse academic” OR “nurse educator” OR “nurse Teacher”) | 372,741 |
| 2 | TI=("chatbot*" OR "chat?bot*" OR "chatter bot*" OR "chatterbot*" OR "chatter robot*" OR "bot*" OR "chat*") AND AB=("chatbot*" OR "chat?bot*" OR "chatter bot*" OR "chatterbot*" OR "chatter robot*" OR "bot*" OR "chat*") | 114,263 |
| 3 | TI=("conversational bot*" OR "conversational agent*" OR "conversational system*" OR "conversational interface*" OR "conversational AI" OR "conversational agenc*" OR "conversational assistant*") AND AB=("conversational bot*" OR "conversational agent*" OR "conversational system*" OR "conversational interface*" OR "conversational AI" OR "conversational agenc*" OR "conversational assistant*") | 1,029 |
| 4 | TI=("smart bot*" OR "smartbot*" OR "smart-bot*") AND AB=("smart bot*" OR "smartbot*" OR "smart-bot*") | 14 |
| 5 | TI=("dialog system*" OR "dialogue system*" OR "dialogue agent*" OR "dialog agent*" OR "digital assistant*") AND AB=("dialog system*" OR "dialogue system*" OR "dialogue agent*" OR "dialog agent*" OR "digital assistant*") | 1,566 |
| 6 | TI=("ai agent*" OR "artificial agent*" OR "assistance technolog*" OR "embodied agent*" OR "relational agent*" OR "virtual agent*" OR "virtual assistant*" OR "virtual coach*" OR "virtual character*" OR "virtual human" OR "intelligent agent*" OR "interactive agent*" OR "artificial intelligence") AND AB=("ai agent*" OR "artificial agent*" OR "assistance technolog*" OR "embodied agent*" OR "relational agent*" OR "virtual agent*" OR "virtual assistant*" OR "virtual coach*" OR "virtual character*" OR "virtual human" OR "intelligent agent*" OR "interactive agent*" OR "artificial intelligence") | 24,327 |
| 7 | TI=("social robot*" OR "virtual advisor*" OR "avatar*" OR "infobot*" OR "animated character*") AND AB=("social robot*" OR "virtual advisor*" OR "avatar*" OR "infobot*" OR "animated character*") | 3,361 |
| 8 | #1 AND (#2 OR #3 OR #4 OR #5 OR #6 OR #7) | 782 |

# **Section 2: Eligibility criteria for study inclusion**

|  | **Inclusion criteria** | **Exclusion criteria** |
| --- | --- | --- |
| Population | Nursers or nursing students including managers, clinical nursers | Doctors, other professional personnel, not human |
| Intervention | Any type of chatbot intervention including, but not restricted to, chatbots apps, messaging, and online website interventions | not specifically focusing on chatbot interventions, no communication with chatbots |
| Comparator | Educate as usual, e.g., face-to-face intervention; drug intervention, no use of chatbot interventions | Comparators that incorporated chatbot intervention at comparable rate with the intervention group but at different frequency |
| Outcomes | Education level or other variables related to education | None |
| Study design | Any | None |
